# Size Matters: How to Characterize ADPKD Severity by Measuring Total Kidney Volume

**DOI:** 10.3390/jcm12186068

**Published:** 2023-09-20

**Authors:** Martin R. Prince, Erin Weiss, Jon D. Blumenfeld

**Affiliations:** 1 Department of Radiology, Weill Cornell Medicine, New York, NY 10065, USA; erw4004@med.cornell.edu; 2 Division of General Medicine, Columbia College of Physicians and Surgeons, New York, NY 10027, USA; 3 Department of Medicine, Weill Cornell Medicine, New York, NY 10065, USA; jdblume@nyp.org; 4 The Rogosin Institute, New York, NY 10065, USA

Following patients with Autosomal Dominant Polycystic Kidney Disease (ADPKD) has been challenging because serum biomarkers such as creatinine often remain normal until relatively late in the disease. The Consortium for Radiologic Imaging Studies of Polycystic Kidney Disease (CRISP) showed that total kidney volume (TKV) can track disease progression before there is any impact on renal function, which makes sense as it reflects the effect of enlarging renal cysts [1]. Mayo Imaging Classification (MIC), based on age and the height-adjusted TKV (ht-TKV), measured at a single time point categorizes patients with diffuse symmetrical kidney cysts into five classes (1A-1E) and rates of disease progression to predict when renal replacement therapy (i.e., dialysis, kidney transplantation) will be required [2]. MIC is used by some to determine eligibility for tolvaptan therapy (i.e., Mayo Classes 1C-1E). Some patients and nephrologists follow TKV regularly to determine if treatment is effective.

What is the best imaging modality for measuring TKV? Ultrasound often has an insufficient field of view to capture an entire kidney that is enlarged by cysts. CT has a sufficient field of view and higher resolution than ultrasound, but cumulative radiation exposure can be significant over a patient’s lifetime. MRI is preferred for measuring TKV because it has a large field of view, high intrinsic image contrast without intravenous enhancement, (e.g., gadolinium), and no ionizing radiation [3].

Despite the advantages of MRI, the precise and reproducible measurement of TKV remains challenging. After obtaining MRI images, how do we optimally extract kidney volumes, and how reliable are those measurements? There are several approaches. The ellipsoidal method of estimating volume (TKV = (length × width × depth × pi/6) is easy to perform and readily available to clinicians who do not have imaging expertise. But this method has poor inter-reader reproducibility, with a coefficient of variation of 7 to 17% [4,5]. Considering that the average annual TKV growth rate in TEMPO 3:4, a clinical trial of tolvaptan treatment in ADPKD, was only 2.8% to 5.5%, with or without tolvaptan treatment, respectively, the ht-TKV measurement reproducibility needs to be well under 2.8% to be useful for annual follow-up assessments. Therefore, the ellipsoidal method is not sufficient.

Manually contouring kidney outlines on every slice of the MRI has better reproducibility, ~3.4% to 6%, but is still not sufficient for monitoring individual subjects [4,5]. Manual contouring is also tedious, time-consuming, and therefore costly because kidney borders on every slice of an MR series of images must be outlined. Typically, an expert radiologist or technologist may require 20 to 30 min for this task. Manual contouring also requires post-processing software to optimize these measurements, which may not be readily available. Another tedious method, known as stereology, is less accurate than manual contouring and has fallen out of favor.

Recently, manual contouring has been partially automated using deep learning methods [6]. Artificial intelligence-based deep learning methods, such as U-NET, outline kidney borders automatically, producing measurements with good inter-reader repeatability between expert observers [6,7,8]. Although this is faster than manual contouring, and some authors claim it to be as precise as the expert radiologist, it does not address the issue of test–retest reproducibility (variability between scans obtained within a short interval where there is no time for organ volume changes), which needs to be less than the annual increase in TKV (2.8–5.5%) [9]. It is not sufficient for measurements only to be as good as the radiologist.

An innovation in MRI measurement of TKV that improves reproducibility and enables the detection of errors in the kidney contouring process [10] solves this dilemma. Deep learning is used to outline kidney contours on all the image sequences that are acquired by a typical MRI study (e.g., T1, T2, and Steady-State Free Precession (SSFP) images), including those in axial, coronal, or sagittal planes. Averaging these multiple TKV measurements reduces variability. Specifically, averaging TKV measurements from five sequences reduced the coefficient of variation between two consecutive exams to 2.5% compared to >5% for measurements performed on only one pulse sequence. Further improvement was achieved by eliminating one outlier and averaging the remaining four measurements. Presumably, this is because there is an error in the outlier measurement which is eliminated by excluding that measurement from the average. Once a sequence was identified as an outlier, it could be corrected and then included in the average. Further investigation into this approach showed that each MRI pulse sequence has intrinsic biases that are mitigated by averaging further enhancing measurement reproducibility [11,12].

Another advantage of deep learning for TKV measurement is its ability to subsegment cysts and renal parenchyma to potentially establish additional biomarkers. For example, exophytic renal cysts that become large because they are not constrained by renal parenchyma can overestimate disease severity. Accordingly, Bae et al. have proposed measuring exophytic cysts separately and excluding them from the TKV measurement [7]. One problem with this approach is deciding which cysts are exophytic. Under their proposed definition, a cyst that is more than 50% beyond the projected outline of the kidney border should be considered exophytic. However, many large cysts are on the borderline between intra-parenchymal and exophytic. To address these limitations, Gregory et al. used deep learning methodology to automatically count the number of cysts and characterize their size and parenchymal surface area, which showed promising improvements in predicting eGFR decline and progression of chronic kidney disease [13]. Yet another important refinement is identifying complex cysts [14]. Renal cysts with hemorrhagic and proteinaceous debris appear bright on T1-weighted images. Counting these T1 bright cysts also improves the accuracy of predicting disease progression. These complex, hemorrhagic cysts are likely more inflammatory and have been associated with a more rapid disease progression [14].

Although MRI measurement of TKV has revolutionized the assessment of the ADPKD phenotype, severity, and prognosis, the methodologic details regarding its measurement are important to ensure optimal reproducibility. Factors such as the characterization of cyst location and content (i.e., exophytic, hemorrhagic, or proteinaceous), blood flow, parenchymal texture, and many other features apparent on MRI, as well as features of this disease in other organs that are near the kidney and, thus, visible on kidney images, may further refine this biomarker for more precise predictions of disease progression [3]. In the meantime, with ADPKD, kidney size is important, and attention towards how TKV is measured can help ensure accuracy and reproducibility.
